# First serological evidence and risk factor analysis of *Neospora caninum* and *Besnoitia besnoiti* infections in cattle and sheep from three regions of Saudi Arabia

**DOI:** 10.14202/vetworld.2025.3229-3242

**Published:** 2025-10-31

**Authors:** Tariq AI-Haboub, Saleh M. Albarrak, Ahmed Elsify, Mosaab A. Omar

**Affiliations:** 1 Department of Pathology and Laboratory Diagnosis, College of Veterinary Medicine, Qassim University, Buraydah, 51452, Saudi Arabia; 2 Department of Animal Medicine and Infectious Diseases, Faculty of Veterinary Medicine, University of Sadat City, Sadat City, Egypt

**Keywords:** *Besnoitia besnoiti*, cattle, enzyme-linked immunosorbent assays, *Neospora caninum*, reproductive health, Saudi Arabia, seroepidemiology, sheep

## Abstract

**Background and Aim:**

Protozoan infections such as *Neospora caninum* and *Besnoitia besnoiti* are significant causes of infertility, abortion, and productivity losses in livestock. Despite their economic impact, epidemiological data from the Kingdom of Saudi Arabia (KSA) remain scarce. This study aimed to determine the seroprevalence of *N. caninum* and *B. besnoiti* in cattle and sheep across Asir, Jeddah, and Al-Qassim regions of KSA and to assess the influence of potential risk factors, including species, sex, age, breed, season, and management system.

**Materials and Methods:**

A cross-sectional study was conducted between June 2024 and March 2025 using 920 serum samples (460 cattle, 460 sheep). Samples were analyzed by indirect enzyme-linked immunosorbent assays using commercial kits (ID *N. caninum* Indirect Screening Kit; ID Screen Besnoitia Indirect 2.0). Associations between seroprevalence and risk factors were evaluated by Chi-square tests at α < 0.05 using Statistical Package for the Social Sciences v23.

**Results:**

The overall seroprevalence of *N. caninum* was 23.91% in cattle and 5.43% in sheep, while *B. besnoiti* antibodies were detected in 5.43% and 3.26%, respectively. Higher seroprevalence was observed in the Asir region, among female animals, and in those aged >1–5 years. The Baladi (cattle) and Daen (sheep) breeds were the most affected. Infections were more frequent during summer and under small-scale or open grazing management. Significant correlations were found between *N. caninum* seroprevalence and both species and sex (p < 0.05), and between *B. besnoiti* seroprevalence and sheep sex (p < 0.05).

**Conclusion:**

This study provides the first comprehensive evidence of *N. caninum* and *B. besnoiti* infections in cattle and sheep in KSA, underscoring their potential economic and reproductive implications. These findings highlight the need for improved biosecurity, control of vector exposure, and regulation of animal movement to mitigate transmission. Future studies should include molecular confirmation and broader geographical coverage to clarify transmission dynamics and genetic diversity of these parasites.

## INTRODUCTION

The Kingdom of Saudi Arabia (KSA) occupies nearly four-fifths of the Arabian Peninsula, spanning approximately 2.15 million km^2^. It is bordered by the Persian Gulf to the east, the Red Sea and Gulf of Aqaba to the west, and Yemen to the south, with land boundaries shared with Jordan, Iraq, and Kuwait in the north and with Qatar, the United Arab Emirates, Oman, and Yemen in the east and south. In addition, Bahrain, an island nation in the Persian Gulf, is linked to the Saudi mainland through a causeway [1].

The total livestock population in KSA is estimated at 25.7 million animals, including camels, cattle, sheep, and goats. Small ruminants dominate this population, comprising approximately 92%, equivalent to about 17.5 million sheep and 6.1 million goats. These species play a vital socioeconomic role in sustaining rural and marginal communities. Meanwhile, the dairy and poultry sectors in KSA operate under intensive, large-scale production systems managed primarily by a limited number of private enterprises [2].

The success and expansion of animal production in any country depend on effective management of infectious diseases, viral, bacterial, fungal, or parasitic, that threaten herd health and productivity. Among these, parasitic diseases remain a major challenge due to the absence of licensed vaccines, limited therapeutic options, emerging drug resistance, globalization, climate change, and a general underestimation of their impact [3].

Protozoan parasites are particularly significant as causes of infertility and abortion in domestic ruminants. *Toxoplasma gondii* and *Sarcocystis* spp. are recognized as major agents of reproductive failure, each possessing a two-host life cycle involving carnivorous definitive hosts that shed infective stages in feces and herbivorous intermediate hosts. The venereally transmitted *Tritrichomonas fetus* also contributes substantially to pregnancy loss in naturally bred cattle worldwide. More recently, *Neospora* spp. has emerged as an important abortifacient in cattle [4], whereas *Besnoitia besnoiti*, the causative agent of bovine besnoitiosis, has re-emerged across Europe and several Asian, African, and Middle Eastern countries. *B. besnoiti* primarily affects cattle, causing severe economic losses due to impaired health, poor productivity, and reproductive inefficiency [5].

*Neospora caninum* is an intracellular protozoan closely related to *T. gondii*, and is now recognized globally as a major pathogen, with canids serving as definitive hosts [6]. Infection with *N. caninum* is associated with reproductive disorders such as abortion in cattle [7] and neonatal mortality in sheep and goats [8–10]. In dogs, the disease manifests as neurological disorders, often with paralysis [7].

In cattle, *N. caninum* persists as a chronic infection that can be vertically transmitted to the fetus, resulting in significant global economic losses. The annual economic burden attributed to *N. caninum* has been estimated at US$1.298–2.380 billion worldwide, with median annual losses of approximately US$1,600 per dairy farm and US$150 per beef farm [11, 12].

Bovine besnoitiosis, caused by the cyst-forming apicomplexan *B. besnoiti*, is a chronic, debilitating, vector-borne disease characterized by cutaneous and systemic lesions. The European Food Safety Authority has classified it as a re-emerging disease due to its increasing incidence and geographic expansion [13]. Clinically, it presents in variable forms, from subclinical to severe, depending on host susceptibility and parasite burden [14]. In endemic herds, most infected cattle remain asymptomatic yet seropositive, while only a minority develops overt clinical signs [13, 15].

A hallmark of *B. besnoiti* infection is the presence of thick-walled tissue cysts in the scleral conjunctiva and vaginal mucosa. The disease causes substantial economic damage through weight loss, decreased milk yield, abortions, transient or permanent infertility in males, and reduced hide value for leather production [13].

Both *N. caninum* and *B. besnoiti* share complex life cycles and are associated with reproductive inefficiency and economic losses in ruminant farms globally [16]. In Saudi Arabia, limited data are available regarding their prevalence. Previous studies have detected *N. caninum* antibodies in various animal species. For instance, *N. caninum* seroprevalence in horses was 10% by competitive-inhibition enzyme-linked immunosorbent assays (ELISA) [17] and 23.9% by Neospora modified agglutination test [18]. Among camels, Aljumaah *et al*. [19] and Mohammed *et al*. [20] reported that seroprevalence rates were 21.99% and 16.6% using ELISA-based methods. However, data on cattle and sheep in KSA remain unavailable, highlighting the need for further epidemiological assessment.

Despite the recognized economic and reproductive impact of *N. caninum* and *B. besnoiti* infections in livestock, epidemiological data from the KSA remain extremely limited. Most previous investigations within the region have focused on *N. caninum* seroprevalence in camels and horses [17–20], whereas information concerning its occurrence in cattle and sheep, the primary sources of meat and dairy production, has not been systematically documented. Furthermore, *B. besnoiti* infection, a re-emerging disease of major concern in Europe and neighboring regions, has not been reported previously in Saudi ruminant populations. This lack of local seroepidemiological data hinders the ability to assess the true burden of these protozoan infections, identify potential risk factors, and develop effective national surveillance and control strategies.

In addition, environmental and management conditions unique to Saudi Arabia, such as its diverse climatic zones (humid coastal to arid inland), widespread smallholder and nomadic livestock systems, and growing intensification of dairy production, may influence the distribution and transmission dynamics of *N. caninum* and *B. besnoiti*. Yet, no comprehensive study has examined how these risk factors interact under Saudi conditions. Consequently, the epidemiological status of these protozoan parasites in the KSA remains poorly understood, creating a critical gap in the One Health understanding of ruminant reproductive diseases in the region.

This study was designed to fill the existing epidemiological and diagnostic gap regarding *N. caninum* and *B. besnoiti* infections in Saudi Arabia. Specifically, it aimed to:

1. Estimate the seroprevalence of *N. caninum* and *B. besnoiti* in cattle and sheep populations from three representative regions of the Kingdom, Asir, Jeddah, and Al-Qassim, using commercial indirect ELISA.
2. Analyze the association between infection and potential risk factors, including species, breed, sex, age, geographical region, season, and management system.
3. Provide baseline data to support future molecular, phylogenetic, and epidemiological studies and to guide the development of national surveillance and control programs targeting protozoan-induced reproductive losses in Saudi livestock.

By addressing these aims, the study contributes essential epidemiological insights that can inform veterinary authorities, policymakers, and farm managers on implementing evidence-based strategies for disease control and livestock productivity enhancement in KSA.

## MATERIALS AND METHODS

### Ethical approval

Ethical approval was granted by the Committee of Research Ethics, Deanship of Scientific Research, Qassim University (Approval No. 18-04-13; December 20, 2023), under the regulations of the National Committee of Bioethics, Saudi Arabia.

### Study period and location

A cross-sectional study was conducted during two distinct seasons of 2024: Winter (Janurary to March) and summer (June to September).

**Figure 1:**
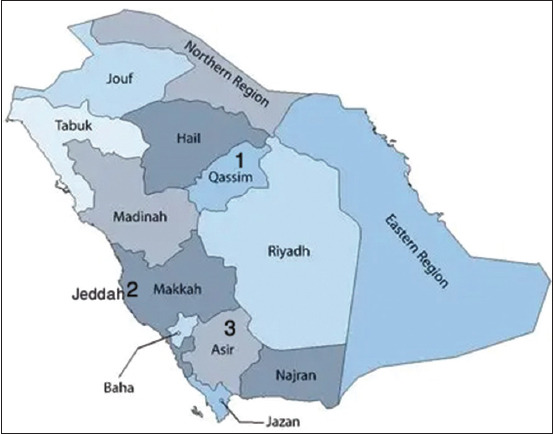
The map of the study area; Numbers indicate the sample collection areas [Source: The map was generated using ArcGIS Pro 3.1 (Esri, Redlands, USA) with regional shapefiles obtained from the GADM database (https://gadm.org/)].

Sampling was carried out in three representative regions of the KSA: Al-Qassim, Jeddah, and Asir (Figure 1).

- Al-Qassim is situated in the central Arabian Peninsula and characterized by cold, wet winters and hot, arid summers.
- Jeddah, located on the Western coast (21.54°N, 39.7°E), experiences a tropical climate – hot and humid summers with mild winters.
- Asir, in the southwestern region (17.25°–19.50°N; 41.50°–50.00°E), has a moderate summer climate (average temperature ≤24°C) and relatively cold winters, making it the coolest of the three regions.

These regions were selected to represent diverse climatic and management conditions across Saudi livestock systems. Table 1 summarizes the distribution of collected samples according to risk factors.

**Table 1:** Number and data of the animals included in the study.

Parameters	Sheep	Cattle
Location		
Al-Qassim	160	170
Jeddah	160	170
Asir	140	120
Sex		
Male	210	210
Female	250	250
Age		
≤1 year	265	315
>1 year to ≥ 5 years	170	75
>5 years	25	70
Season		
Winter	220	170
Summer	240	290
Management system	170	
Individual rearing	120	
Small-scale rearing		
Intensive-scale rearing		160
Open grazing		300
Cattle breed		
Foreign	195	
Baladi	265	
Sheep breeds		
Daen		210
Naemy		165
Foreign		85

All assays were performed at the Department of Pathology and Laboratory Diagnosis, College of Veterinary Medicine, Qassim University.

### Animal population and sampling

The minimum sample size was estimated using the single population proportion formula:


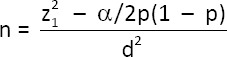


Where n is the required sample size, Z = 1.96 (for 95% confidence), p = expected prevalence (assumed 0.5 in the absence of prior data), and d = allowable error (0.05). This approach yields the maximum possible sample size, ensuring adequate statistical power [21–23]. The calculated minimum was 384, with additional samples included to compensate for potential data loss, resulting in a final total of 920 animals.

Blood samples were collected from 460 cattle and 460 sheep, all apparently healthy and unvaccinated against *Neospora* or *Besnoitia*. Approximately 5 mL of blood was drawn aseptically from the jugular vein into plain tubes (without anticoagulant). Samples were left to clot for 30 min, refrigerated for 3 h to contract the clot, and centrifuged at 378 × g for 5 min at room temperature. Serum was collected using sterile Pasteur pipettes and stored at −20°C until serological analysis.

### Serological analysis

Serological screening for antibodies against *N. caninum* and *B. besnoiti* was performed using commercial indirect ELISA Kits (ID Vet, France).

- *N. caninum* was detected using the ID *N. caninum* Indirect Screening Kit (Product Code: NCS; Batch No. P64-0323).
- *B. besnoiti* antibodies were detected using the ID Screen Besnoitia Indirect 2.0 Kit (Product Code: BSNTB; Batch No. 034).

Both assays were carried out according to the manufacturer’s instructions.

### *N. caninum* ELISA procedure

The ID *N. caninum* Indirect Screening Kit detects ruminant immunoglobulins against *N. caninum* in serum, plasma, or milk using an anti-ruminant Ig conjugate. Serum samples and controls were diluted 1:10. The manufacturer-reported performance parameters were 100% sensitivity (95% confidence interval [CI]: 98.8%–100%) and 100% specificity (95% CI: 99.63%–100%).

A test was considered valid when:

- The mean optical density (OD) of the positive control (OD_p_C) >0.350, and
- The ratio (OD_p_C/O_n_C) >3.

Sample positivity was expressed as a percentage (S/P %), calculated as:


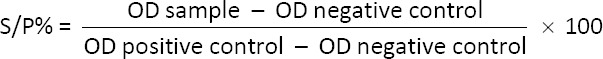


### Interpretation

- S/P ≤ 40% → Negative
- 40% < S/P < 50% → Doubtful
- S/P ≥ 50% → Positive.

Samples with doubtful results were excluded from statistical analysis.

### *B. besnoiti* ELISA procedure

The ID Screen Besnoitia Indirect 2.0 Kit was validated in an inter-laboratory study, demonstrating 97.2% sensitivity and 100% specificity [15]. Serum samples and controls were diluted 1:10 and analyzed in duplicate on 96-well plates. Each sample was tested in two adjacent wells: one coated with *B. besnoiti* purified antigen (even columns) and one uncoated (odd columns). Corrected OD values were obtained by subtracting the uncoated well OD from the coated well OD.

The assay was considered valid when the positive control OD > 0.350 and the OD ratio between positive and negative controls >3. The S/P % was calculated as:


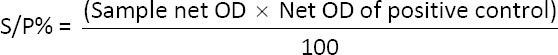


### Interpretation

- S/P < 25% → Negative
- 25%–30% → Uncertain
- ≥30% → Positive.

Doubtful samples were excluded from further analysis.

### OD measurement

OD readings were obtained at 450 nm using a BIOTEK EL808 microplate reader (Serial No. 1301301C) with Gen5™ Microplate Reader and Imager Software (BioTek Instruments, USA).

### Statistical analysis

All collected data were entered into Microsoft Excel 365 (Microsoft Office, Washington, USA) spreadsheets and analyzed using Statistical Package for the Social Sciences v23 (IBM Corp., USA). Seroprevalence was calculated as the proportion of positive samples among all tested samples. Associations between seroprevalence and risk factors (species, sex, breed, age, location, season, and management system) were evaluated using the Chi-square (χ^2^) test at a 95% confidence level, with α < 0.05 considered statistically significant.

## RESULTS

### Seroprevalence of *N. caninum*

The overall seroprevalence of *N. caninum* among the examined animals is summarized in Table 2. Antibodies against *N. caninum* were detected in 23.91% of cattle and 5.43% of sheep. Statistical analysis revealed a significant difference between species (χ^2^ = 16.75, df = 2, p < 0.05), indicating that cattle were more frequently infected than sheep.

**Table 2:** Neospora *caninum* seroprevalence.

Risk factors examined	Total examined	Doubtful		Negative		Positive	
		
		No.	Percentage	No.	Percentage	No.	Percentage
Cattle							
Breeds	460	110	23.91	315	68.47	35	7.60
Baladi	265	80	30.18	175	66.03	10	3.77
Foreign	195	30	15.38	140	71.79	25	12.82
Sex							
Male	210	35	16.66	170	80.95	5	2.3
Female	250	75	30	145	58	30	12
Age							
≤1 year	315	65	20.63	215	68.25	35	11.11
>1 year to ≥ 5 years	75	30	40	45	60	0	0
>5 years	70	15	21.42	55	78.57	0	0
Location							
Al-Qassim	170	35	20.58	125	73.52	10	5.88
Jeddah	170	40	23.52	110	64.70	20	11.76
Asir	120	35	29.16	80	66.66	5	4.16
Season							
Summer	290	85	29.31	175	60.34	30	10.34
Winter	170	25	14.70	140	82.35	5	2.94
Management system							
Individual rearing	170	45	26.47	110	64.70	15	8.82
Small-scale rearing	120	40	33.3	65	54.16	15	12.5
Intensive-scale rearing	170	25	14.7	140	82.35	5	2.94
Sheep							
Breeds	460	25	5.43	425	92.39	10	2.17
Daen	210	10	4.76	195	92.85	5	2.38
Naemy	165	10	6.06	150	90.90	5	3.03
Foreign	85	5	5.88	80	94.11	0	0
Sex							
Male	210	10	4.76	195	92.85	5	2.38
Female	250	15	6	230	92	5	2
Age							
≤1 year	265	15	5.66	245	92.45	5	1.88
>1 year to ≥ 5 years	170	10	5.88	155	91.17	5	2.94
>5 years	25	0	0	25	100	0	0
Location							
Al-Qassim	160	10	7.14	145	89.28	5	3.57
Jeddah	160	5	3.12	155	96.87	0	0
Asir	140	10	6.25	125	90.62	5	3.12
Season							
Summer	240	15	6.25	220	91.66	5	2.08
Winter	220	10	4.54	205	93.18	5	2.27
Management system							
Open grazing	300	20	6.66	270	90	10	3.33
Intensive-scale rearing	160	5	3.12	155	96.87	0	0

### Breed-wise distribution

Among cattle, seroprevalence varied between breeds, with 30.18% in the Baladi breed and 15.38% in foreign breeds. In sheep, the rates were 4.76% in Daen, 6.06% in Naemy, and 5.88% in foreign breeds. However, there was no statistically significant association between breed and *N. caninum* infection for either species (cattle: χ^2^ = 4.58, df = 2; sheep: χ^2^ = 0.56, df = 4; p > 0.05).

### Sex-wise distribution

Sex-based analysis revealed that female cattle had a higher seroprevalence (30%) compared to male cattle (16.66%). Similarly, ewes (6%) showed slightly higher rates than rams (4.76%). A significant correlation was detected between infection and sex in cattle (χ^2^ = 6.22, df = 2, p < 0.05), while the association was not significant in sheep (χ^2^ = 0.08, df = 2, p > 0.05).

### Age-related distribution

Animals were categorized into three age groups: ≤1 year, >1–5 years, and >5 years. In cattle, seroprevalence rates were 20.63%, 40%, and 21.42%, respectively. In sheep, rates were 5.66%, 5.88%, and 0%, respectively. Despite the apparent increase in infection among cattle aged >1–5 years, no significant association was found between *N. caninum* seroprevalence and age in either species (cattle: χ^2^ = 0.54; sheep: χ^2^ = 0.54; df = 4; p > 0.05).

### Geographical distribution

Regional variation was observed among the three study locations. In cattle, the seroprevalence rates were 29.16% in Asir, 23.52% in Jeddah, and 20.58% in Al-Qassim. In sheep, rates were 6.25%, 3.12%, and 7.14%, respectively. Although the highest prevalence was recorded in the Asir region, the difference among locations was not statistically significant (cattle: χ^2^ = 5.72; sheep: χ^2^ = 1.69; df = 4; p > 0.05).

### Seasonal variation

Seasonal analysis revealed a higher seroprevalence of *N. caninum* during the summer compared to winter. In cattle, infection rates were 29.31% in summer and 14.70% in winter, while in sheep, they were 6.25% and 4.54%, respectively. The difference between seasons was not statistically significant (cattle: χ^2^ = 4.97; sheep: χ^2^ = 0.13; df = 2; p > 0.05).

### Effect of the management system

Management practices appeared to influence the infection pattern. The highest seroprevalence in cattle (33.3%) was recorded in a small-scale rearing system, whereas the lowest (14.7%) was recorded in an intensive system. Among sheep, open grazing systems had higher infection rates (6.66%) than intensive systems (3.12%). However, the associations between rearing systems and *N. caninum* infection were not statistically significant (cattle: χ^2^ = 5.72; sheep: χ^2^ = 1.65; df = 4; p > 0.05).

### Seroprevalence of *B. besnoiti*

The overall results of *B. besnoiti* infection and its relationship with risk factors are summarized in Table 3. The seroprevalence of *B. besnoiti* was 5.43% in cattle and 3.26% in sheep, with no significant difference between species (χ² = 0.52, df = 2, p > 0.05).

**Table 3:** Besnoitia *besnoiti* seroprevalence.

Risk factors examined	Total examined	Doubtful		Negative		Positive	
		
		No.	Percentage	No.	Percentage	No.	Percentage
Cattle							
Breeds	460	25	5.43	425	92.39	10	2.17
Baladi	265	20	7.54	245	92.45	0	0
Foreign	195	5	2.56	180	92.30	10	5.12
Sex							
Male	210	5	2.38	205	97.61	0	0
Female	250	20	8.00	220	88	10	4
Age							
≤1 year	315	10	3.17	300	95.23	5	1.58
>1 year to ≥ 5 years	75	10	13.33	65	86.66	0	0
>5 years	70	5	7.14	60	97.05	5	7.14
Location							
Asir	120	20	16.66	100	83.33	0	0
Jeddah	170	5	2.94	160	94.11	5	2.94
Al-Qassim	170	0	0	165	97.05	5	2.94
Season							
Summer	290	25	8.62	260	89.65	5	1.72
Winter	170	0	0	165	97.05	5	2.94
Management system							
Individual rearing	170	5	2.94	160	94.11	5	2.94
Small-scale rearing	120	20	16.66	100	83.33	0	0
Intensive-scale rearing	170	0	0	165	97,05	5	2.94
Sheep							
Breeds	460	15	3.26	435	94.56	10	2.17
Daen	210	10	4.76	195	92.85	5	2.83
Naemy	165	0	0	165	100	0	0
Foreign	85	5	5.88	75	88.23	5	5.88
Sex							
Male	210	5	2.38	200	95.23	5	2.38
Female	250	10	4	235	94	5	2
Age							
≤1 year	265	5	1.88	255	96.22	5	1.88
>1 year to ≥5 years	170	10	5.88	155	91.17	5	2.94
>5 years	25	0	0	25	100	0	0
Location							
Asir	160	10	6.25	150	93.75	0	0
Jeddah	160	0	0	155	96.87	5	3.12
Al-Qassim	140	5	3.57	130	92.85	5	3.57
Season							
Summer	240	10	4.16	225	93.75	5	2.08
Winter	220	5	2.38	210	9545	5	2.27
Management system							
Open grazing	300	15	5	280	93.33	5	1.66
Intensive-scale rearing	160	0	0	155	96.87	5	3.12

### Breed-wise distribution

In cattle, the seroprevalence rates were 7.54% in the Baladi breed and 2.56% in foreign breeds. Among sheep, rates were 4.76% in Daen, 0% in Naemy, and 5.88% in foreign breeds. Breed had no significant association with infection in either species (cattle: χ^2^ = 1.18; sheep: χ^2^ = 3.53; p > 0.05).

### Sex-wise distribution

Female animals exhibited a higher prevalence of *B. besnoiti* antibodies than males. In cattle, 8% of cows and 2.38% of bulls were seropositive. In sheep, 4% of ewes and 2.38% of rams tested positive. However, the observed differences were not statistically significant (cattle: χ^2^ = 3.21; sheep: χ^2^ = 0.19; p > 0.05).

### Age-related distribution

Seroprevalence increased with age up to mid-life and then declined. In cattle, infection rates were 3.17% (≤1 year), 13.33% (>1–5 years), and 7.14% (>5 years). In sheep, the corresponding rates were 1.88%, 5.88%, and 0%, respectively. The differences among age groups were not statistically significant (cattle: χ^2^ = 4.51; sheep: χ^2^ = 1.70; df = 4; p > 0.05).

### Geographical distribution

Regional variation was observed, with the highest *B. besnoiti* prevalence in Asir (16.66% in cattle and 6.25% in sheep), followed by Jeddah (2.94% in cattle and 0% in sheep), and Al-Qassim (0% in cattle and 3.57% in sheep). Despite these differences, statistical analysis showed no significant correlation between infection and location (cattle: χ^2^ = 8.32; sheep: χ^2^ = 3.02; df = 4; p > 0.05).

### Seasonal variation

Higher *B. besnoiti* seroprevalence was detected in summer than in winter. In cattle, infection rates were 8.62% and 0%, respectively, while in sheep, they were 4.16% and 2.38%. These differences were not statistically significant (cattle: χ^2^ = 3.14; sheep: χ^2^ = 0.26; p > 0.05).

### Effect of management system

The management system also influenced *B. besnoiti* exposure. Cattle raised under small-scale rearing systems exhibited the highest prevalence (16.66%), whereas intensive systems showed no infection (0%). Among sheep, the open grazing system showed higher infection (5%) compared to intensive systems (0%). Nevertheless, these differences were not statistically significant (cattle: χ^2^ = 8.32; sheep: χ^2^ = 1.80; df = 4; p > 0.05).

## DISCUSSION

### Infectious disease control context

Infectious disease control programs and strategies depend on many factors. Identifying the causative pathogens and determining their prevalence, along with analyzing the risk factors contributing to their spread, are the first essential steps in any control program. These data enable veterinary authorities to make concise and evidence-based decisions.

Despite the relevance of *N. caninum* and *B. besnoiti*, there are no records or updated data regarding their occurrence among cattle and sheep in KSA. This represents a major challenge to improving the livestock industry. Neosporosis and besnoitiosis are real threats to the cattle and sheep industries and adversely affect the national economy due to the severe health hazards they induce.

### Zoonotic and epidemiological aspects

No viable *N. caninum* has been isolated from human tissues; thus, little is known about the epidemiological and zoonotic aspects of *N. caninum* infection in humans. However, as this parasite has multiple intermediate hosts, it is possible for humans to become infected [24]. *B. besnoiti* is not a zoonotic pathogen, which means that it does not easily spread to people. The parasite that causes bovine besnoitiosis primarily infects cattle and other ruminants, and it is thought that *B. besnoiti* is incapable of infecting humans [25].

### Seroprevalence of *N. caninum*

#### Comparative seroprevalence across regions

The seroprevalence of *N. caninum* among the cattle under investigation was 23.91%, which is similar to that recorded by Ibrahim *et al*. [26] in the Egyptian Delta (20.43%) and by Metwally *et al*. [27] in Upper Egypt (24.6%). However, it was higher than that recorded by Jilo Tache *et al*. [28] in southern Ethiopia (5%) and by Koiwai *et al*. [29] in Japan (5.7%). Moreover, it was lower than that reported by Abdeltif *et al*. [30] in Algeria (36.2%) and Kasap *et al*. [31] in Turkey (33.3%).

The seroprevalence of *N. caninum* in cattle fluctuates based on location, age, sex, and breed, as well as on the serological test employed. Prior research indicated prevalence rates ranging from 7.6% to 97.2% in America, 3.9%–24.1% in Africa, 0.5%–60% in Asia, 0.7%–76% in Europe, and 3.2%–46.7% in Australia [28].

#### Prevalence in sheep and comparison with other countries

Among the examined sheep, the seroprevalence rate was 5.43%. This rate is similar to those reported in central China (7.3% and 8.4%) and Egypt (8.6%). In contrast, it was lower than the rates reported in Northwest Spain, the Czech Republic, Egypt, Italy, São Paulo (Brazil), Minas Gerais (Brazil), and Northern Jordan (10.1%, 12.2%, 36.1%, and 25.6%, respectively). It was, however, higher than those recorded in Turkey (2.1%), Australia (2.2%), and Slovakia (3.7%) [32]. The variance in disease prevalence may be attributed to diverse regional or ecological factors, sheep breeds, rearing practices, or the timing and methodology of surveys [33].

#### Geographical distribution within Saudi Arabia

The three examined areas had different seroprevalence rates for *N. caninum*. For cattle, the seroprevalence rates were 29.16%, 23.52%, and 20.58% in Asir, Jeddah, and Al-Qassim, respectively. For sheep, the rates were 6.25%, 3.12%, and 7.14%, respectively. Statistically, there was no significant correlation between location and seroprevalence rate. This variation is consistent with the findings of Cao *et al*. [34], who reported that the seroprevalence of *N. caninum* in cattle differs significantly across nations, and even among areas and farms within the same country.

#### Influence of host factors

In this investigation, the seroprevalence of *N. caninum* was significantly higher among cattle than among sheep (Table 2). This trend was also reported by Liu *et al*. [35], Gharekhani *et al*. [36], and Fereig *et al*. [37]. Female animals (both cattle and sheep) had significantly higher seroprevalence than male animals (Table 2), which agrees with studies of Metwally *et al*. [27], Olmo *et al*. [38], and Nooruldeen *et al*. [39]. They reported that female cattle had a higher prevalence rate than male cattle. Furthermore, Selim *et al*. [32] and Wang *et al*. [40] reported that ewes had higher seroprevalence than rams. This is potentially connected to differing hormone levels between rams and ewes, as well as stress factors related to pregnancy and lactation [41].

#### Age and breed association

According to statistical analysis, no significant correlation was found between seroprevalence rate and animal age (for both cattle and sheep). This finding aligns with investigations from Brazil, Croatia, Jordan, Romania, and Venezuela, which revealed no correlation between animal age and *N. caninum* seroprevalence, suggesting that vertical transmission is likely more significant in these flocks [37, 42]. However, Wei *et al*. [43] observed a significant connection between age and *N. caninum* seroprevalence in cattle in China, where older cattle are more likely to have been exposed to infective oocysts. In this study, animals aged >1–5 years showed higher prevalence rates.

Local cattle and sheep breeds (Daen and Naemy) exhibited higher seroprevalence rates of *N. caninum* than foreign breeds. Similar findings were reported by Semango *et al*. [33] among cattle in Tanzania. Statistical analysis also revealed no significant correlation between *N. caninum* seroprevalence and breed, a finding confirmed by Noori *et al*. [44], who noted that most studies have found no breed correlation and that infection rates are primarily influenced44 by management factors.

#### Seasonal and management influences

The summer season showed a higher prevalence rate than the winter for both cattle and sheep, although the correlation was not statistically significant. This observation is in agreement with studies of Selim *et al*. [32] and Mohammed and A’aiz [45].

Cattle from small-scale rearing farms had the highest seroprevalence rate, whereas those in intensive rearing systems had the lowest. Sheep reared on open grazing had higher prevalence rates than those in intensive systems. For both species, there was no significant correlation between rearing system and *N. caninum* seroprevalence. These findings agree with those of Jilo *et al*. [28], who reported a high seroprevalence in small rearing systems, although [34] found higher rates in extensive ranch systems.

The high prevalence may be related to management and biosecurity issues, such as the introduction of untested animals, the absence of biosecurity, or dog access to placental and fetal tissues. Such conditions promote congenital transmission and sustain infection within herds [46].

### Seroprevalence of *B. besnoiti*

#### First detection in Saudi Arabia

This study represents the first evidence of *B. besnoiti* infection in cattle and sheep in KSA. The seroprevalence among cattle was 5.43%, similar to the 5.7% recorded in Malaysia [17] and 6% in Jordan [47], but lower than the 22.1% and 10.6% reported in Egypt [37, 48]. Among sheep, the seroprevalence was 3.26%, comparable to 4.68% in Spain [49], 3.21% in China [50], and 3.3% in Egypt [37]. Statistically, there was no significant effect of animal species on the prevalence of protozoa. The higher prevalence in cattle than sheep observed here is consistent with Fereig *et al*. [37] and Oryan *et al*. [51].

#### Regional and breed variation

According to geographical region, Asir had a high seroprevalence of *B. besnoiti* infection (16.66% in cattle and 6.25% in sheep), followed by Jeddah (2.94% and 0%) and Al-Qassim (0% and 3.57%). The prevalence of *B. besnoiti* infection varies greatly across studies. Observed variations may arise from differences in breed composition, serologic methods, cutoff values, management systems, degrees of animal contact, and vector exposure [47].

No significant correlation was found between animal breed and seroprevalence. Baladi cattle had 7.54% positivity compared to 2.56% in foreign breeds. For sheep, the rates were 4.76%, 0%, and 5.88% for Daen, Naemy, and foreign breeds, respectively. This variation suggests potential genetic influences that require further investigation [27].

#### Effect of sex and age

Female animals had a higher prevalence of *B. besnoiti* infection than males in both species. Although the relationship between sex and prevalence was not significant in cattle, it was significant in sheep. Similar findings were reported in Spain [52], China [50, 53, 54], and Egypt [47], where infection was 3% in females and absent in males. Conversely, Dubey *et al*. [24] and Gutiérrez-Expósito *et al*. [49] reported higher prevalence in males than females, and Gazzonis *et al*. [55] found a higher infection risk in males (60%) compared to females (38.8%) in Italy.

The higher infection rate in females may be attributed to greater exposure or susceptibility due to reproductive stress-related immune suppression [52, 53, 55]. In addition, infection may be transmitted to females through copulation with infected males [56].

High prevalence rates were recorded in animals aged between 1 and 5 years, which is consistent with previous studies by Fereig *et al*. [37], Talafha *et al*. [47], Fernández-García *et al*. [52], Ashmawy and Abu-Akkada [53], Álvarez-García *et al*. [57], and Anastácio *et al*. [58]. The increased rate in older animals supports the role of horizontal transmission and prolonged exposure in the dissemination of parasites [59].

#### Seasonal and management influences

The prevalence rate of *B. besnoiti* was higher in summer than in winter for both species, though the association was not significant. This contrasts with the findings of Kuraa *et al*. [48], who observed a higher winter prevalence; however, our findings are biologically plausible, as infection rates rise with vector activity (e.g., horseflies) during hot seasons [60].

Although not statistically significant, *B. besnoiti* infection was most common in small-scale cattle systems (16.66%) and in open-grazing sheep (5%), while intensive rearing systems recorded 0%. Many authors have linked higher prevalence to management and hygiene factors [47, 48, 55]. Elevated infection on farms may stem from persistent exposure, natural mating, and insect-borne transmission, as well as direct contact between infected and naïve animals, facilitating spread [47, 48, 55].

## CONCLUSION

This study provides the first comprehensive serological evidence of *N. caninum* and *B. besnoiti* infections in cattle and sheep populations from three distinct regions of the Kingdom of Saudi Arabia. Overall, *N. caninum* antibodies were detected in 23.91% of cattle and 5.43% of sheep, while *B. besnoiti* antibodies were found in 5.43% of cattle and 3.26% of sheep. The seroprevalence of *N. caninum* was significantly higher in cattle than in sheep, and infection was notably associated with sex, with females showing higher rates than males. In contrast, no significant associations were found between *B. besnoiti* infection and most of the studied risk factors, although a higher seroprevalence was observed in the Asir region, in animals aged 1–5 years, and during the summer season. Management systems influenced infection rates, with small-scale and open-grazing setups showing comparatively higher prevalence than intensive rearing systems.

The detection of *N. caninum* and *B. besnoiti* in Saudi livestock highlights an underestimated reproductive health threat with potential economic repercussions for the national cattle and sheep industries. The results emphasize the need for targeted biosecurity measures, including restricting dog access to pastures and calving areas to interrupt *N. caninum* transmission cycles, enhancing hygienic handling of fetal membranes and aborted materials to minimize horizontal spread, implementing serological screening before herd introductions to prevent disease introduction, and strengthening vector control programs, particularly during summer, to reduce *B. besnoiti* transmission through biting flies. These findings provide actionable epidemiological data for policymakers, veterinarians, and producers to design control strategies tailored to small-scale and extensive rearing systems where infection pressure is highest.

This study’s strengths include its large sample size (920 animals), multi-species approach, and regional coverage across three major Saudi climatic zones, enabling a comparative understanding of parasite distribution under varying ecological and management conditions. Furthermore, the use of validated commercial ELISA kits with high sensitivity and specificity ensures the reliability of serological outcomes.

Despite these strengths, several limitations should be acknowledged. Serological detection indicates exposure but not active infection; hence, it does not distinguish between current and past infections. Molecular confirmation (polymerase chain reaction) and parasite isolation were not performed, limiting the ability to confirm circulating strains or link infection to clinical reproductive losses. Seasonal sampling, though representative, may not fully capture temporal variation in transmission cycles. The absence of farm-level reproductive and production data constrained the assessment of the parasites’ direct economic impact.

Future investigations should integrate molecular and histopathological confirmation to validate serological findings and explore the genetic diversity of circulating *Neospora* and *Besnoitia* strains in Saudi Arabia. Longitudinal and case–control studies linking infection to abortion, infertility, and production losses are crucial to establish true epidemiological and economic burdens. Furthermore, exploring the potential role of wildlife reservoirs and canid populations in parasite maintenance would enhance understanding of transmission ecology. The inclusion of vector ecology studies could also elucidate the role of insect-borne spread under Saudi climatic conditions.

In summary, this pioneering study confirms the presence and circulation of *N. caninum* and *B. besnoiti* among cattle and sheep in Saudi Arabia, providing a foundational epidemiological baseline for national surveillance and control. The observed infection patterns underscore the urgent need for increased awareness, enhanced biosecurity measures, and integrated parasite management within livestock systems. Establishing a national monitoring program and strengthening diagnostic capacity will be pivotal to mitigate reproductive losses, safeguard animal productivity, and enhance the overall sustainability of the Saudi livestock sector.

## AUTHORS’ CONTRIBUTIONS

MO, AE, and SA: Conceptualized the study and revised the manuscript. TA: Investigation and data collection. TA and AE: Data analysis and revised the manuscript. AE: Drafted the manuscript. All authors have read and approved the final version of the manuscript.
